# Mr. Geoghagan on Hernia

**Published:** 1831-01-01

**Authors:** 


### MR. GEOGHAGAN ON HERNIA.

In the year 1811, Mr. Geoghagan, a surgeon of some eminence in Dublin, published some observations on strangulated hernia in the Edinburgh Medical and Surgical Journal, in which he advocated the propriety of cold applications, bleeding, and other means, before proceeding immediately to the taxis—on the principle, or, at least, the hypothesis that inflammation was the cause of the stricture or strangulation.

"The surgeon (says he) who is impressed with the notion, that he is to cure *inflamed hernia,* will never place his patient on his head, and toss him about; such practice may increase, but cannot abate the tumefaction, which is the *sine qua non;* he will not attempt to push it by force through an aperture which bears no proportion to its dimension. No: he will proceed as in all cases of visceral inflammation, enjoin composure, and hold firmly in his view, that UNTIL THE OBSTRUCTION IN THE INTESTINE, which is the effect of inflammation, is removed, its replacement will not cure, and ought not to be attempted. He will return the hernia after the inflammatory stage, when it can be done with safety, as curative of hernia, but not of strangulation, as it is termed. That the operation has very often terminated fatally, although less frequently than formerly, must be acknowledged. The cause assigned for this termination has generally been, that it was performed too late, to which I assent, in a great measure; but I particularly attribute it to a mistaken view of the disease, and the practice founded upon that view. It is represented as if entangled, and to be disengaged by jolting the entire body, and forcing with the finger and entire hand, &c. &c. which is by no means calculated to remove the contents (although it does so sometimes per accident) and must aggravate the symptoms so as to render future operation unavailing. I consider the jolting as improper as it would be in enteritis. The French surgeons succeed more frequently than we do, which is attributed to operating early: I attribute their success to bleeding, which they carry to great extent, and to their avoiding manual efforts very much. Desault forbid them altogether; indeed their chief reliance is on bleeding. The many cases recorded, my own experience and view of the disease, warrant me in believing, that if the hands were not applied, but strict attention paid to composure and position, and bleeding particularly attended to, that the operation would be less frequently necessary and oftener successful. I conduct the manipulation in the manner an elastic bottle or a bladder is emptied when administering an enema. It is last in my order of treatment before the knife; first with every one else. The representation of all our modern writers, that the gut is girt as if bound by a cord, strongly opposes their own practice, as to the taxis, by supposing an immoveable state, which every touch of the fingers must inflame, and still further enlarge, whilst the indication is to diminish."

More recently Mr. G. has published a letter to Mr. Abernethy on the same subject. In this letter he reiterates his conviction that the strangulation consists in an "impervious gut, *pro*

*tempore*, caused by inflammation, confining the contents, which, being highly pungent, and stagnant, greatly aggravate the symptoms." The usual pressure of the tumour so inflamed against stiff tendinous borders, he observes, irritates the gut, favours derangement of structure, and produces adhesion, thus effecting permanent obliteration of the tube, when this state has continued long.

